# Inhibiting the inhibitors: Development of the IAP inhibitor xevinapant for the treatment of locally advanced squamous cell carcinoma of the head and neck

**DOI:** 10.1016/j.ctrv.2022.102492

**Published:** 2022-11-30

**Authors:** Robert L. Ferris, Kevin Harrington, Jonathan D. Schoenfeld, Makoto Tahara, Christina Esdar, Satu Salmio, Andreas Schroeder, Jean Bourhis

**Affiliations:** a University of Pittsburgh Medical Center, Pittsburgh, PA, USA; b The Royal Marsden NHS Foundation Trust, London, UK; c Dana-Farber Cancer Institute, Boston, MA, USA; d National Cancer Center Hospital East, Kashiwa, Chiba Prefecture, Japan; e Merck Healthcare KGaA, Darmstadt, Germany; f Centre Hospitalier Universitaire Vaudois, Lausanne, Switzerland

**Keywords:** Locally advanced squamous cell carcinoma of the head and neck, Apoptosis, Chemoradiotherapy, Inhibitor of apoptosis protein, Xevinapant

## Abstract

Standard of care for patients with locally advanced squamous cell carcinoma of the head and neck (LA SCCHN) is surgery followed by chemoradiotherapy (CRT) or definitive CRT. However, approximately 50 % of patients with LA SCCHN develop disease recurrence or metastasis within 2 years of completing treatment, and the outcome for these patients is poor. Despite this, the current treatment landscape for LA SCCHN has remained relatively unchanged for more than 2 decades, and novel treatment options are urgently required. One of the key causes of disease recurrence is treatment resistance, which commonly occurs due to cancer cells’ ability to evade apoptosis. Evasion of apoptosis has been in part attributed to the overexpression of inhibitor of apoptosis proteins (IAPs). IAPs, including X-linked IAP (XIAP) and cellular IAP 1 and 2 (cIAP1/2), are a class of proteins that regulate apoptosis induced by intrinsic and extrinsic apoptotic pathways. IAPs have been shown to be overexpressed in SCCHN, are associated with poor clinical outcomes, and are, therefore, a rational therapeutic target. To date, several IAP inhibitors have been investigated; however, only xevinapant, a potent, oral, small-molecule IAP inhibitor, has shown clinical proof of concept when combined with CRT. Specifically, xevinapant demonstrated superior efficacy in combination with CRT vs placebo + CRT in a randomized, double-blind, phase 2 trial in patients with unresected LA SCCHN. Here, we describe the current treatment landscape in LA SCCHN and provide the rationale for targeting IAPs and the clinical data reported for xevinapant.

## Introduction

Head and neck cancer, which includes cancers of the oral cavity, larynx, nasopharynx, oropharynx, and hypopharynx, is the eighth most common cancer worldwide, with 878,348 new cases and 444,347 deaths reported in 2020 [1]. In Europe, 158,581 cases and 69,328 deaths were reported in 2020, and 24,093 cases and 8,240 deaths were reported in Japan [2]. In the US, it is estimated that 66,470 new cases and 15,050 deaths occurred in 2022 [3,4].

Squamous cell carcinoma of the head and neck (SCCHN) accounts for>90 % of all head and neck cancers [5]. Tobacco use, frequent alcohol intake, and human papillomavirus infection are 3 factors that increase the risk of developing SCCHN [6–8]. Most patients (≈60 %) with SCCHN are diagnosed with locally advanced (LA) disease [9]. Of these patients, approximately half will not undergo surgery [10] due to the presence of inoperable tumors, physician decision to avoid surgery in order to pre-serve organ function/quality of life, or patient preference [5].

## Treatment landscape for LA SCCHN

The current standard of care for patients with unresected LA SCCHN who are eligible to receive cisplatin is definitive chemoradiotherapy (CRT), ie, concurrent high-dose cisplatin and radiotherapy: cisplatin 100 mg/m^2^ every 3 weeks (3 cycles) plus standard fractionated radiotherapy 70 Gy in 35 daily fractions of 2 Gy over 7 weeks [5,11]. In patients who will undergo surgery and are at a high risk of disease recurrence, the standard of care is surgery plus adjuvant CRT [5,11]. However, patients may have an absolute contraindication for the use of cisplatin [12] or physicians might prefer not to use cisplatin in patients with a relative contraindication to cisplatin [12]. In patients at high risk of recurrence following surgery in whom cisplatin may not be preferred, the recommended treatment is radiotherapy alone [5,11,12]. Outcomes for this subset of patients are poor with a high rate of disease recurrence [13,14].

### Unresected LA SCCHN

The treatment landscape for unresected LA SCCHN has remained relatively unchanged for more than 2 decades. One area of clinical development for the treatment of unresected LA SCCHN has been the study of immune checkpoint inhibitors (ICIs) in combination with CRT. ICIs are approved in the recurrent and/or metastatic SCCHN setting [15,16]; however, they have not improved outcomes in unresected LA SCCHN. In the JAVELIN Head and Neck 100 phase 3 trial, the addition of avelumab (anti–programmed cell death 1 ligand 1 [anti–PD-L1]) to CRT did not improve progression-free survival (PFS) vs placebo + CRT in patients with unresected LA SCCHN [17]. Similarly, the phase 3 KEYNOTE-412 trial, which evaluated pembrolizumab (anti–programmed cell death 1 protein [anti–PD-1]) + CRT vs placebo + CRT followed by maintenance pembrolizumab or placebo in patients with unresected LA SCCHN, did not meet its primary endpoint of improving event-free survival [18]. Additionally, the phase 3 REACH trial in patients with unresected LA SCCHN, which evaluated the combination of avelumab + cetuximab + radiotherapy vs CRT in cisplatin-eligible patients or cetuximab + radiotherapy in cisplatin-ineligible patients, did not meet the primary endpoint of improving PFS in cisplatin-ineligible patients, and favored standard-of-care CRT for cisplatin-eligible patients [19]. Lastly, the phase 2 PembroRad study, investigating pembrolizumab + radiotherapy in patients with unresected LA SCCHN unfit to receive cisplatin, did not improve outcomes vs cetuximab + radiotherapy [20]. Currently, the phase 3 IMvoke010 trial is investigating atezolizumab (anti–PD-L1) as treatment for patients with LA SCCHN who are at high risk of disease recurrence or progression following definitive local therapy [21]. The NANORAY-312 phase 3 trial is also investigating the combination of the hafnium dioxide nanoparticle radioenhancer NBTXR3 + radiotherapy with or without cetuximab vs radiotherapy with or without cetuximab in platinum-ineligible, elderly patients with unresected LA SCCHN [22].

### Resected LA SCCHN

Similar to the trial landscape of unresected LA SCCHN, a number of clinical studies that are investigating the use of ICIs in combination with CRT are ongoing for the treatment of patients who will undergo surgery. A phase 3 trial, KEYNOTE-689, is investigating neoadjuvant pembrolizumab followed by surgical resection and adjuvant pembrolizumab + radiotherapy or CRT [23]. The phase 3 NIVOPOSTOP trial is investigating nivolumab (anti–PD-1) + CRT vs CRT alone in patients with resected LA SCCHN who are at high risk of relapse [24], while the phase 3 IMvoke010 trial is investigating atezolizumab as adjuvant therapy for patients with LA SCCHN who are at high risk of disease recurrence or progression following primary surgery that was performed as part of definitive therapy [21]. Additionally, a phase 3 trial is investigating nimotuzumab (anti–epidermal growth factor receptor) + CRT vs CRT alone as adjuvant therapy for patients with resected LA SCCHN [25].

Due to the poor long-term outcomes in patients with unresected LA SCCHN who experience local disease recurrence or distant metastasis and the high risk of relapse in patients who undergo surgery but are ineligible for cisplatin-based adjuvant CRT, novel treatment options are urgently required.

## Inhibitor of apoptosis proteins (IAPs)

Resistance to chemotherapy and/or radiotherapy is commonly observed in cancer and is one of the key factors in local or distant failure [26]. Apoptosis is often suppressed in cancer cells, and evasion of apoptosis represents a key hallmark of cancer [27,28], enabling cancer cells to resist the effects of chemotherapy/radiotherapy [29–31]. One important mechanism of suppression of apoptosis and resistance to anticancer therapy has been attributed to IAPs [30–32].

**Fig. 1.**
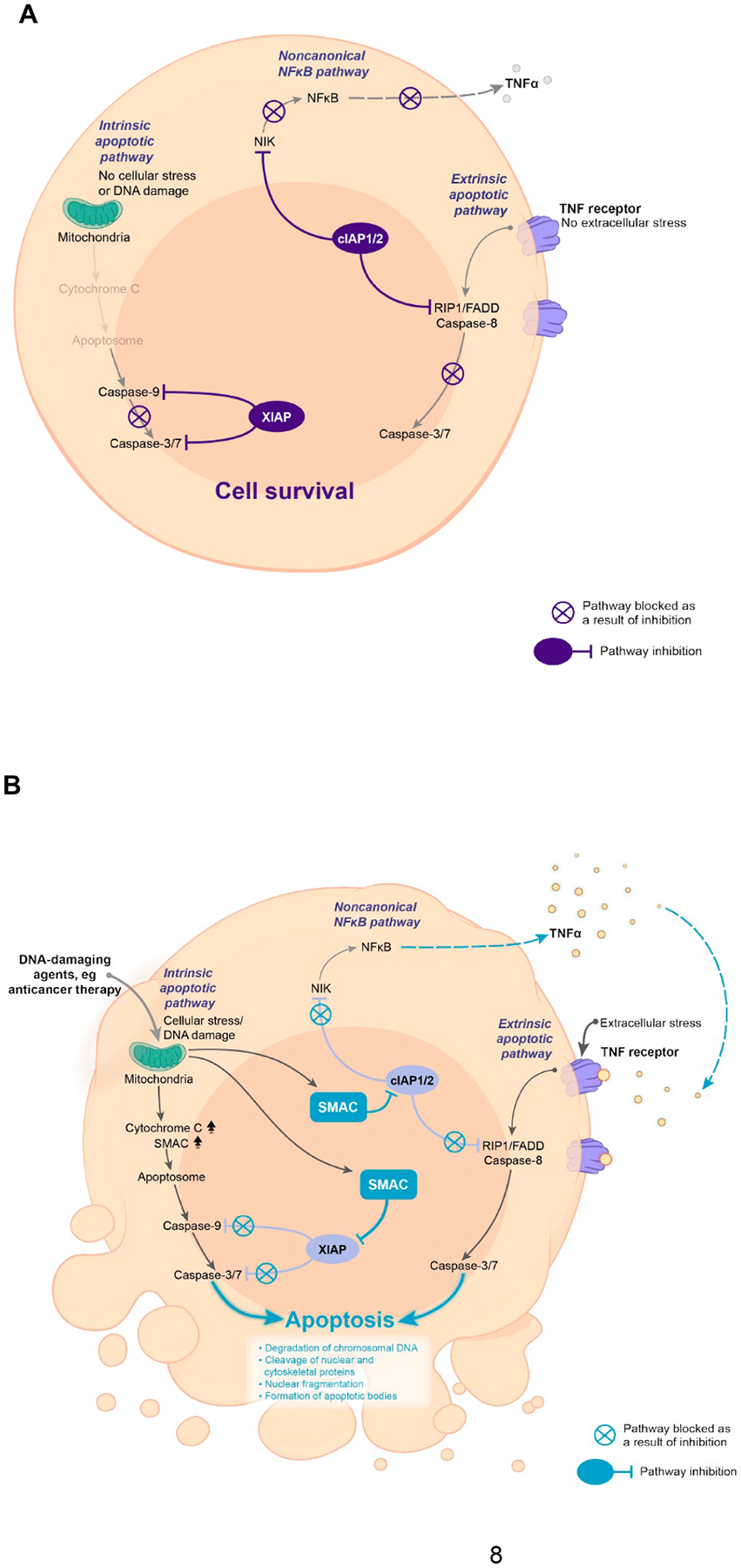
Regulation of apoptotic signaling in healthy cells. (A) IAPs block apoptotic signaling pathways, promoting prosurvival signaling. XIAP and cIAP1/2 prevent activation of caspases and, thereby, suppress apoptosis; cIAP1/2 also blocks NIK activity and thereby inhibits noncanonical NFκB signaling, suppressing the release of inflammatory cytokines, such as TNFα. (B) SMAC inhibits IAPs, promoting apoptosis. SMAC is released from the mitochondria into the cytosol in response to proapoptotic stimuli. SMAC inhibits XIAP and cIAP1/2, enabling the activation of the caspase cascade and promoting downstream apoptotic signaling and production of inflammatory cytokines via the noncanonical NFκB pathway. cIAP1/2, cellular IAP 1/2; FADD, fas-associated protein with death domain; IAP, inhibitor of apoptosis proteins; NFκB, nuclear factor kappa-light-chain enhancer of activated B cells; NIK, NFκB-inducing kinase; RIP1, receptor-interacting serine/threonine-protein kinase 1; SMAC, second mitochondria-derived activator of caspase; TNF, tumor necrosis factor; XIAP, X-linked IAP.

IAPs, including X-linked IAP (XIAP) and cellular IAP 1 and 2 (cIAP1/2), are a class of proteins that regulate apoptosis induced by intrinsic and extrinsic apoptotic pathways [33–35]. IAPs block apoptotic signaling through a variety of different mechanisms (Fig. 1); XIAP directly blocks caspase activity downstream of the mitochondrion (intrinsic apoptotic pathway) by binding and inhibiting caspases-3, −7, and −9 [34,35], while cIAP1/2 inhibits the formation of proapoptotic complexes that are part of the extrinsic apoptotic pathway initiated by tumor necrosis factor (TNF) receptor signaling [33,34,36,37].

In addition, cIAP1/2 also activates canonical and noncanonical nuclear factor kappa-light-chain enhancer of activated B cells (NFκB) signaling, which can modulate apoptosis and immune signaling. cIAP1/2 also regulates apoptosis via ubiquitination of receptor-interacting serine/threonine-protein kinase 1 (RIP1), which initiates canonical NFκB signaling through the recruitment of transforming growth factor-β (TGF-β)-activated kinase 1-TAK1-binding protein, IκB kinase, and NF-kappa-B essential modulator, and leads to the degradation of IκB and release of NFκB, activating the transcription of genes involved in prosurvival signaling [38,39]. cIAPs suppress noncanonical NFκB signaling by ubiquitination of NFκB-inducing kinase (NIK), preventing activation of the noncanonical pathway and suppressing the release of inflammatory cytokines such as TNFα [39–43].

Activity of IAPs is inhibited by endogenous antagonist proteins such as the second mitochondria-derived activator of caspase (SMAC), released from the mitochondria in response to intrinsic stress, eg, induced by anticancer therapy, leading to apoptosis [33,34,44]. SMAC binds to the baculoviral IAP repeat domains of XIAP and cIAP1/2 [45], preventing them from inhibiting the activation of downstream caspases, and promoting apoptotic signaling [33,44]. The binding of SMAC to cIAP1/2 also results in the ubiquitination and degradation of cIAP and activation of the extrinsic apoptotic pathway [33,38]. In addition, the reduction in cIAP1 results in the stabilization of NIK, enabling the activation of noncanonical NFκB signaling and inducing TNFα expression [33,46], which in turn can induce apoptosis via the extrinsic pathway [38].

IAPs, including XIAP and cIAP1/2, have been shown to be frequently overexpressed in various cancers [34,38], including SCCHN [47], and increase the resistance of cancer cells to apoptosis and prevent cell death induced by anticancer treatments, such as chemotherapy and radiotherapy [31,32,48,49]. XIAP expression was also significantly associated with cisplatin resistance and poor clinical outcomes in LA SCCHN [50]. In addition, increased XIAP/cIAP1 expression and cIAP2 overexpression have been associated with a poor prognosis in SCCHN [50–52]. Due to the link between IAP overexpression and tumor progression, treatment failure, and poor prognosis, targeting of IAPs is considered a promising therapeutic concept in LA SCCHN.

## IAP inhibitors

To explore the potential of IAP inhibition for the treatment of cancer, a number of IAP inhibitors have been developed. IAP inhibitors are generally SMAC mimetics, designed to bind and inhibit XIAP and cIAP1/2 [33,38]. IAP proteins have also been inhibited using antisense oligonucleotides that downregulate protein levels by targeting their messenger RNAs [34]. IAP inhibitors have shown antitumor activity in cell lines and mouse xenograft models, especially in combination with other anticancer therapies [53–59]. However, despite recent advances in this area of research, the majority of IAP inhibitors are in the preliminary stages of clinical development or have been discontinued (Table 1).

## Development of IAP inhibitors in LA SCCHN

Currently, 4 IAP inhibitors are under investigation in clinical trials in SCCHN (Table 1), including xevinapant, tolinapant, APG-1387, and birinapant, of which 2 are being investigated in LA SCCHN. Three trials are currently ongoing in LA SCCHN: a phase 1 trial of tolinapant in combination with radiotherapy in cisplatin-ineligible patients with unresected disease (no data have been published to date); a phase 3 trial of xevinapant in combination with CRT in patients with unresected disease; and a phase 3 trial of xevinapant in combination with radiotherapy in the adjuvant postoperative setting in cisplatin-ineligible patients. Of these inhibitors, xevinapant is the most advanced in terms of clinical development and, to date, the only IAP inhibitor that has demonstrated antitumor activity and proof of concept in unresected LA SCCHN in a randomized, placebo-controlled, double-blind, phase 2 clinical trial [60].

## Xevinapant

**Fig. 2.**
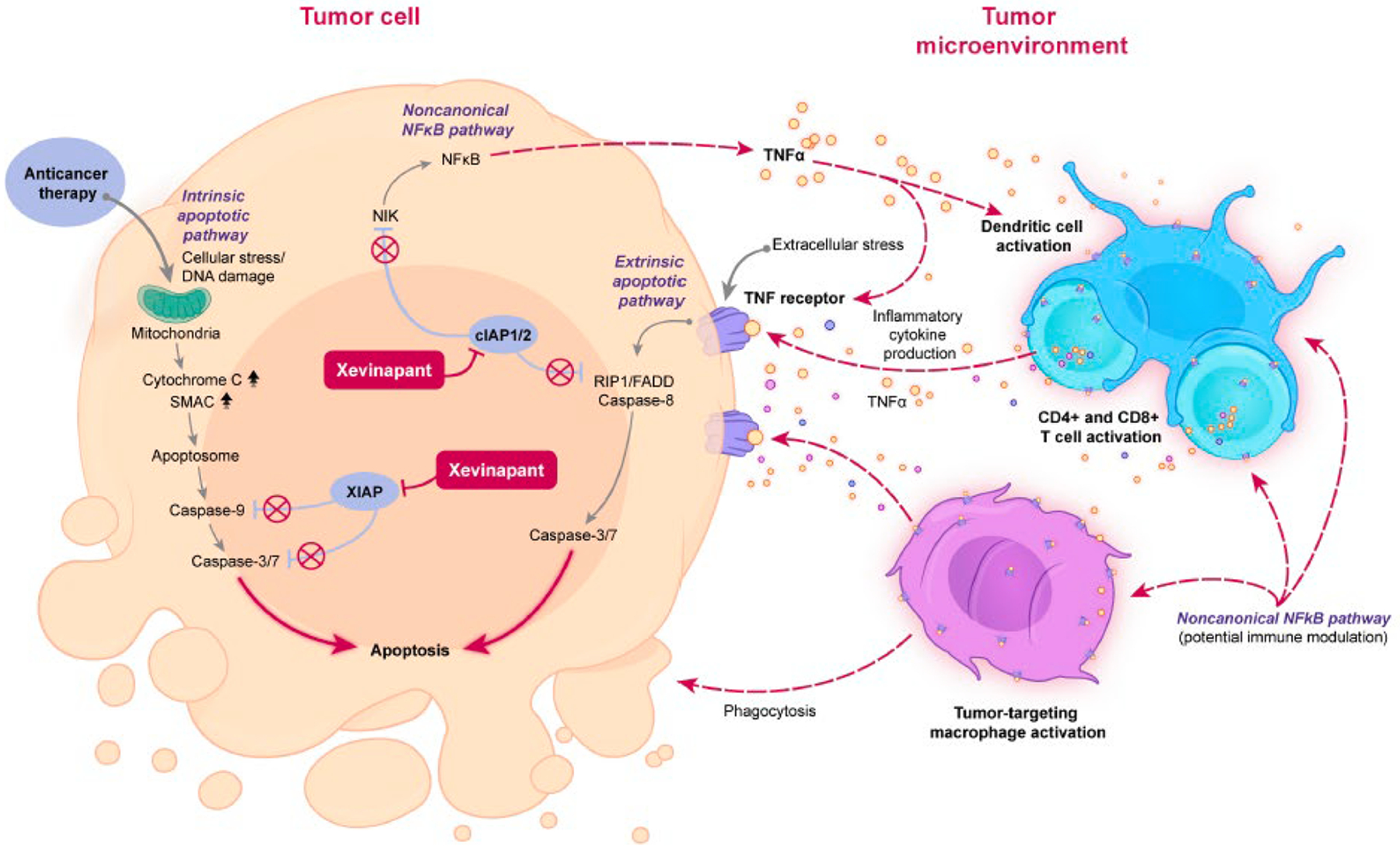
Xevinapant mode of action. Xevinapant is a first-in-class, oral, small-molecule IAP inhibitor. It is thought to (A) restore apoptosis in cancer cells by blocking XIAP and cIAP1/2, releasing caspase activity downstream of the intrinsic mitochondrial and the extrinsic TNF receptor pathways, and (B) enhance the inflammatory antitumor response in immune cells of the tumor microenvironment by activating noncanonical NFκB signaling through blocking of cIAP1/2 downstream of the TNF receptor (potential effect based on the known function of cIAP1/2 in the control of NFκB signaling). CD, cluster of differentiation; cIAP1/2, cellular IAPs 1 and 2; FADD, fas-associated protein with death domain; IAP, inhibitor of apoptosis proteins; NFκB, nuclear factor kappa-light-chain-enhancer of activated B cells; NIK, NFκB-inducing kinase; RIP1, receptor-interacting serine/threonine kinase 1; SMAC, second mitochondria-derived activator of caspase; TNFα, tumor necrosis factor alpha; XIAP, X-linked IAP.

Xevinapant is a potent, oral, small-molecule IAP inhibitor that blocks XIAP and cIAP1/2, restoring cancer cell sensitivity to apoptosis and, thereby, is thought to enhance the effects of anticancer treatments such as chemotherapy and radiotherapy [61–63]. Xevinapant acts as a SMAC mimetic and releases the blockade in downstream caspase activity crucial for apoptosis [62]; inhibition of XIAP directly releases the brake on downstream caspase activity in the intrinsic pathway [62], while inhibition of cIAP1/2 promotes proapoptotic signaling from TNF receptors via the extrinsic pathway [33,62] (Fig. 2). In tumor samples from patients with SCCHN, xevinapant + cisplatin or carboplatin induced caspase-3–dependent apoptosis [64]. In SCCHN cell lines and mouse xenograft models, xevinapant exhibited limited activity as a single agent and synergistic/additive activity with chemotherapy and radiotherapy [62,65].

In addition, the inhibition of cIAP1/2 by xevinapant may amplify immune cell activation by activating noncanonical NFκB signaling, which induces the production of inflammatory cytokines, such as TNFα [36,62,66–68]. Through the activation of noncanonical NFκB signaling and production of inflammatory cytokines, xevinapant is expected to promote the activation of B cells, T cells, and macrophages, enhancing cytokine secretion and upregulating immune activation markers [36,69–71]. Preclinical studies of the IAP inhibitors tolinapant and LCL161 support this hypothesis; IAP inhibition by tolinapant in a mouse model of SCCHN enhanced clonal expansion of cytotoxic T cells, improved tumor-infiltrating lymphocyte–mediated killing of tumor cells, and upregulated antigen presentation on tumor cells [72]. Additionally, the modulation of noncanonical NFκB signaling through cIAP1/2 inhibition by LCL161 in a mouse model of pancreatic cancer enhanced macrophage activation, increasing phagocytosis of tumor cells in a T-cell–dependent manner [73].

Consequently, the overexpression of IAPs in SCCHN and the potential for xevinapant to enhance the effect of chemotherapy and radiotherapy provide a strong rationale for the exploration of xevinapant treatment in patients with LA SCCHN.

## Xevinapant clinical data

The combination of xevinapant + CRT was explored in a phase 1/2 trial in patients with unresected LA SCCHN. In the phase 1, open-label, dose-escalation part of the trial, patients received escalating doses of xevinapant (100, 200, or 300 mg/day on days 1–14 of a 3-week cycle, for 3 cycles) combined with concurrent CRT (cisplatin 100 mg/m^2^ on day 2 every 3 weeks, for 3 cycles, with concomitant conventional fractionated radiotherapy [2 Gy/day, 5 days/week for 7 weeks] up to 70 Gy) [74]. A predictable and manageable safety profile was observed at the maximum tolerated dose of xevinapant 200 mg/day, which was defined as the recommended phase 2 dose [74]. Across all dose levels, confirmed objective responses were observed in 11 of 13 evaluable patients (85 %) [74]. In the double-blind, phase 2 part of the trial, a total of 96 patients with unresected LA SCCHN were randomly assigned to receive xevinapant 200 mg/day (days 1–14 of 3-week cycles, for 3 cycles) + CRT (cisplatin 100 mg/m^2^ every 3 weeks on day 2 of every cycle, for 3 cycles; intensity-modulated radiotherapy 70 Gy [2 Gy/day, 5 days/week for 7 weeks]) or placebo + CRT [60]. At 18 months from the end of CRT, a significantly larger proportion of patients achieved locoregional control (primary endpoint) with xevinapant + CRT (54 %; 95 % CI, 39–69) vs placebo + CRT (33 %; 95 % CI, 20–48; odds ratio, 2.74; 95 % CI, 1.15–6.53; *P* = 0.0232) [60,75]. Over 3 years of follow-up, xevinapant + CRT prolonged PFS and duration of response vs placebo + CRT: median PFS was not reached vs 16.9 months (hazard ratio, 0.33; 95 % CI, 0.17–0.67; *P* = 0.0019), the risk of death or disease progression after initial response was reduced by 79 % (hazard ratio, 0.21; 95 % CI, 0.08–0.54; *P* = 0.0011) [75]. The risk of death over 5 years of follow-up was more than halved with xevinapant + CRT vs placebo + CRT (hazard ratio, 0.47; 95 % CI, 0.27–0.84; *P* = 0.0101) [75]. In summary, results from the phase 1/2 trial of xevinapant + CRT in patients with unresected LA SCCHN suggest the addition of xevinapant to standard-of-care CRT is well tolerated and results in superior clinical outcomes vs placebo + CRT.

In a window-of-opportunity study in patients with resectable SCCHN, treatment with xevinapant monotherapy resulted in a significant reduction in cIAP1 levels in tumor cells [67]. This study also provided support for the downstream effects on host immunity in the tumor microenvironment [67]. A significant increase in levels of cluster of differentiation 8^+^ (CD8^+^) tumor-infiltrating lymphocytes and PD-1/PD-L1–positive immune cells in patient tumor samples was observed after xevinapant treatment and, in addition, changes in expression of genes related to NFκB signaling were identified [67].

## Future outlook

Xevinapant is currently being investigated in 2 randomized, placebo-controlled, double-blind, phase 3 studies. The TrilynX study is evaluating xevinapant + CRT vs placebo + CRT in approximately 700 patients with unresected LA SCCHN, with event-free survival as the primary endpoint of the study [76]. The XRay Vision study is evaluating xevinapant + radiotherapy vs placebo + radiotherapy in approximately 700 patients with resected LA SCCHN who are at a high risk of disease recurrence and are ineligible to receive cisplatin, with disease-free survival as the primary endpoint [77].

## Conclusion

Long-term outcomes are poor for patients with unresected LA SCCHN and patients with resected disease who are at a high risk of disease recurrence. Currently, in the wake of recent failures in trials adding ICIs to CRT, there are relatively few clinical studies in this disease area and novel treatment options are urgently required. IAP inhibitors are an emerging therapeutic class for LA SCCHN due to overexpression of IAPs in this tumor type and the role of IAPs in resistance to standard-of-care anticancer therapies. Preclinical data for xevinapant provided a strong rationale for the inhibition of IAPs in combination with CRT or radiotherapy in LA SCCHN, and the phase 2 study of xevinapant + CRT vs placebo + CRT was the first randomized trial in decades to show clinical improvement vs standard of care in unresected LA SCCHN. Phase 3, randomized, placebo-controlled, double-blind studies evaluating xevinapant in patients with LA SCCHN are ongoing.

## Figures and Tables

**Table 1:** Active listings of IAP inhibitors registered on ClinicalTrials.gov.

Agent	Active listings on ClinicalTrials.gov*	Phase	Type of treatment	Tumor type(s)	Status	NCT number
*AEG35156*	No	–	–	–	–	–
AEG40826 (HGS1029)	No	–	–	–	–	–
*APG-1387 (SM-1387)*	Yes (3)	1	Monotherapy or in combination with pembrolizumab or carboplatin/paclitaxel	Advanced solid tumors or hematologic malignancies	Recruiting	NCT03386526
		1/2	+ toripalimab	Advanced solid tumors, phase 2 nasopharyngeal cohort	Recruiting	NCT04284488
		1/2	+ chemotherapy	Pancreatic adenocarcinoma	Recruiting	NCT04643405
*BI891065*	Yes (1)	1	+ BI754091	Asian patients with advanced solid tumors	Active, not recruiting	NCT04138823
*Birinapant (TL32711)*	Yes (2)	1	+ IMRT	Locally recurrent SCCHN	Recruiting	NCT03803774
		1	+ IGM-8444	Advanced solid tumors	Recruiting	NCT04553692
*BV6*	No	–	–	–	–	–
*CUDC-427 (GDC-0917)*	No	–	–	–	–	–
*JP1201*	No	–	–	–	–	–
*LBW242*	No	–	–	–	–	–
*LCL161*	No	–	–	–	–	–
*RG7419 (GDC-0152)*	No	–	–	–	–	–
*SM114*	No	–	–	–	–	–
*SM130*	No	–	–	–	–	–
*SM164*	No	–	–	–	–	–
*Tolinapant (ASTX660)*	Yes (4)	1	+ pembrolizumab	Cervical, TNBC, advanced cancer	Recruiting	NCT05082259
		1/2	Monotherapy	Advanced solid tumors and lymphomas, phase 2 recurrent/metastatic SCCHN cohort	Active, not recruiting	NCT02503423
		1/2	Monotherapy	Relapsed/refractory T-cell lymphoma	Active, not recruiting	NCT04362007
		1	+ radiotherapy	Unresected LA SCCHN (cisplatin ineligible)	Recruiting	NCT05245682
*Xevinapant (Debio 1143)*	Yes (3)	1	+ pembrolizumab	Advanced/metastatic pancreatic and colorectal adenocarcinoma	Active, not recruiting	NCT03871959
		3	+ CRT	Unresected LA SCCHN	Recruiting	NCT04459715
		3	+ IMRT	Resected LA SCCHN (cisplatin ineligible)	Recruiting	NCT05386550

CRT, chemoradiotherapy; IMRT, intensity-modulated radiation therapy; LA, locally advanced; SCCHN, squamous cell carcinoma of the head and neck; TNBC, triple-negative breast cancer.

* Active listings include trials with the status: not yet recruiting; recruiting; or active, not recruiting.
